# Unsupervised semantic label generation in agricultural fields

**DOI:** 10.3389/frobt.2025.1548143

**Published:** 2025-02-25

**Authors:** Gianmarco Roggiolani, Julius Rückin, Marija Popović, Jens Behley, Cyrill Stachniss

**Affiliations:** ^1^ Center for Robotics, University of Bonn, Bonn, Germany; ^2^ Micro Air Vehicle Laboratory, Delft University of Technology, Delft, Netherlands; ^3^ Lamarr Institute for Machine Learning and Artificial Intelligence, Dortmund, Germany

**Keywords:** agricultural automation, robotic crop monitoring, deep learning for agricultural robots, semantic scene understanding, automatic labeling, unsupervised learning

## Abstract

Robust perception systems allow farm robots to recognize weeds and vegetation, enabling the selective application of fertilizers and herbicides to mitigate the environmental impact of traditional agricultural practices. Today’s perception systems typically rely on deep learning to interpret sensor data for tasks such as distinguishing soil, crops, and weeds. These approaches usually require substantial amounts of manually labeled training data, which is often time-consuming and requires domain expertise. This paper aims to reduce this limitation and propose an automated labeling pipeline for crop-weed semantic image segmentation in managed agricultural fields. It allows the training of deep learning models without or with only limited manual labeling of images. Our system uses RGB images recorded with unmanned aerial or ground robots operating in the field to produce semantic labels exploiting the field row structure for spatially consistent labeling. We use the rows previously detected to identify multiple crop rows, reducing labeling errors and improving consistency. We further reduce labeling errors by assigning an “unknown” class to challenging-to-segment vegetation. We use evidential deep learning because it provides predictions uncertainty estimates that we use to refine and improve our predictions. In this way, the evidential deep learning assigns high uncertainty to the weed class, as it is often less represented in the training data, allowing us to use the uncertainty to correct the semantic predictions. Experimental results suggest that our approach outperforms general-purpose labeling methods applied to crop fields by a large margin and domain-specific approaches on multiple fields and crop species. Using our generated labels to train deep learning models boosts our prediction performance on previously unseen fields with respect to unseen crop species, growth stages, or different lighting conditions. We obtain an IoU of 88.6% on crops, and 22.7% on weeds for a managed field of sugarbeets, where fully supervised methods have 83.4% on crops and 33.5% on weeds and other unsupervised domain-specific methods get 54.6% on crops and 11.2% on weeds. Finally, our method allows fine-tuning models trained in a fully supervised fashion to improve their performance in unseen field conditions up to +17.6% in mean IoU without additional manual labeling.

## 1 Introduction

The demand for food is constantly increasing due to the growing world population, requiring new methods to optimize crop production (Horrigan et al., 2002; Ewert et al., 2023; Storm et al., 2024; Walter et al., 2017). The use of robotic systems in agriculture has the promise to make processes, such as monitoring fields (Ahmadi et al., 2020; Boatswain Jacques et al., 2021), phenotyping (Weyler et al., 2022b), and weed spraying (Wu et al., 2020), more efficient and sustainable (Cheng et al., 2023). Commonly, robotic platforms perceive their environment using deep learning methods to semantically interpret complex data collected with onboard sensors (Dainelli et al., 2024). However, these approaches usually require large amounts of human-labeled data to achieve satisfactory performance for real-world deployment and often fall short in unseen field conditions (Wang et al., 2022; Magistri et al., 2023).

In this paper, we examine the problem of automated semantic crop-weed segmentation in RGB images, enabling robots to perform tasks, such as automated weeding (Balabantaray et al., 2024; Saqib et al., 2023), controlled usage of pesticide (Murugan et al., 2020), harvesting (Pan et al., 2023), or phenotyping (Weyler et al., 2022a). We aim to maximize a semantic segmentation neural network’s performance in various field deployment conditions, e.g., different growth stages, crop species, or lighting conditions, without human-labeled training data. This is crucial to ensure a robust crop-weed segmentation in new unseen fields to enable robots to perform weeding and harvesting. Our approach automatically labels onboard RGB images based on the robot’s pose and the current map of the field semantically segmented into crops and weeds. In this way, semantic labels are generated using the robot’s spatial information and the field arrangement’s crop row structure.

Previous heuristic-based methods for unsupervised semantic segmentation in agriculture proposed by Lottes et al. (2017) and Winterhalter et al. (2018) rely on poorly generalizing assumptions about field arrangements, e.g., absence of weeds in the crop row (Lottes et al., 2017), constant distance between plants’ rows (Lottes and Stachniss, 2017; Winterhalter et al., 2018), or non-overlapping vegetation components (Lottes et al., 2017). Although fully supervised deep learning-based approaches do not rely on geometric assumptions, they rely on in-domain human-labeled training data. The performance of such approaches is satisfactory when being deployed in conditions similar to those they were trained on. However, their performance usually rapidly deteriorates in novel deployment conditions, e.g., different crop species, weeds pressure, lighting conditions, or growth stage, requiring new human-labeled training data. This is costly and makes these approaches impractical for application when there is not enough time, money, or data to re-train the approach on new field conditions.

**FIGURE 1:**
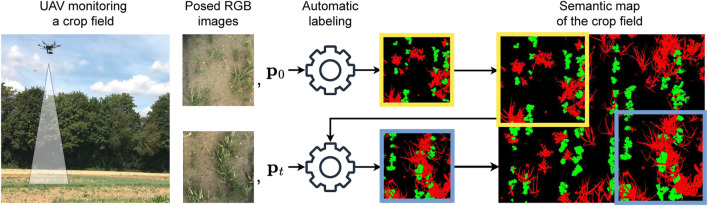
The overview of our pipeline to generate semantic labels for images of crop fields. We use a robotic platform equipped with an RGB camera to collect posed images of the field. Each image gets processed by our automatic labeling method, generating a semantic segmentation of the image to fuse into the semantic map. At each time step, we use the current semantic map to generate the image’s semantic label and update the map accordingly.

The main contribution of this paper is a novel heuristic approach for unsupervised soil-weed-crop segmentation in managed agricultural fields addressing these limitations. Our method automatically generates labels used to train deep semantic segmentation networks. The overview of our pipeline is shown in Figure 1. Our pipeline takes the current RGB image and the camera pose of the robotic platform as input to compute a semantic map of the field. As a key novelty, we use the semantic map to enforce the spatial consistency of labels. To this end, we propagate the information about the crop rows in the map, leading to better crop segmentation across different growth stages. Additionally, we do not assign labels to vegetation components that are close to the crop rows but are not classified as crops. This reduces possible labeling errors and thus improves model predictions after training on our generated labels. We use the generated image-label pairs to train an uncertainty-aware evidential semantic segmentation network (Sensoy et al., 2018). At inference, as a post-processing step, we exploit the predicted uncertainties to refine the final semantic predictions.

In sum, we make three key claims: our approach (i) generates more accurate semantic labels than previous unsupervised label generation approaches on multiple crop species, growth stages, and lighting conditions; (ii) we outperform previous unsupervised semantic segmentation approaches by combining our spatially consistent generated labels and uncertainty-aware semantic neural networks; and (iii) improve performance of fully supervised models on previously unseen crops, growth stages, or soil conditions after fine-tuning using our automatically generated labels. These claims are backed up by our experimental evaluation. We open-source our code upon paper acceptance.

## 2 Related work

Our work uses heuristic-based computer vision techniques for semantic segmentation of RGB images to automatically generate weed-crop segmentation labels of agricultural fields for training a semantic segmentation network. We train the network in an uncertainty-aware fashion using evidential deep learning (Sensoy et al., 2018) to post-process predictions at inference time based on their uncertainty.

### 2.1 Heuristic-based semantic segmentation

Otsu (1979) proposed using gray-level histograms for binary image segmentation based on an automatic threshold assuming a bimodal distribution for fore- and background pixels. Pong et al. (1984) propose the region-growing algorithm segmenting images in multiple regions after providing initial seeds for each region. Similarly, the Watershed algorithm (Najman and Schmitt, 1996) requires user-defined markers to segment objects using a distance function. To overcome the need for initial seeds, Canny (1986) used edge detectors to distinguish regions. To incorporate statistical image features for segmentation, Loyd (1982) adopted the K-means algorithm. To allow automatic robotic intervention in the fields, Riehle et al. (2020) and Gao et al. (2020) applied semantic segmentation techniques to the agricultural domain. Lottes et al. (2017) further advance these general-purpose approaches by exploiting the field arrangement and deploying their method on a ground field robot. Similarly, our approach also exploits the field arrangement to automatically segment images. In contrast, we additionally enhance spatial label consistency using robotic semantic mapping. Further, we do not assign labels to image parts likely to include labeling errors. In this way, we reduce the number of erroneous crop and weed instances, which is essential to achieve high prediction performance and consistent uncertainty estimation of the trained deep neural network.

### 2.2 Learning-based semantic segmentation

Recent approaches mainly use neural networks to extract latent image features for semantic segmentation. Various convolutional neural network architectures (Romera et al., 2018; He et al., 2017), and more recently, Vision transformers (Strudel et al., 2021; Cao et al., 2023) have been applied to semantic segmentation. A large portion of these approaches have also been evaluated or adapted to the agricultural domain. Cui et al. (2024) propose an improvement to the U-net architecture by Ronneberger et al. (2015) to segment corns and weeds while Zenkl et al. (2022) use the DeepLabV3 architecture by Chen et al. (2017) to segment wheat. These approaches usually require vast amounts of per-pixel human-labeled training data, covering all the desired crop species, growth stages, lighting conditions, and other deployment conditions to ensure promising test-time performance. Hence, many works have investigated how to reduce the labeling effort of deep learning-based approaches. One popular method is pre-training the network on different easy-to-label tasks, e.g., image classification (Deng et al., 2009) or using self-supervision (Chen et al., 2020), and fine-tuning the pre-trained network using few human-labeled per-pixel annotations specific to the target application. Other works propose to train networks on sparse labels instead of dense per-pixel labels (Lee et al., 2022), so called weakly supervised semantic segmentation. In the agricultural domain, Zhao et al. (2023) reduce the need for per-pixel labels using scrawl annotations, i.e., manually drawn lines, to weakly supervise a semantic segmentation model. Chen et al. (2024) remove per-pixel annotations completely, only exploiting reference images to localize disease symptoms in plants, using an innovative class activation mapping method. In contrast to Chen et al. (2024), we propose a new unsupervised approach to automatically generate per-pixel semantic segmentation labels exploiting domain knowledge of the field arrangement. Our semantic labels can be directly used for network training without the need for human labels or for fine-tuning pre-trained networks on unseen fields.

### 2.3 Uncertainty-aware deep learning

Classical neural networks are known to often provide overconfident wrong point estimate predictions (Abdar et al., 2021). Several works, including the one by Lakshminarayanan et al. (2017), use ensembles of multiple independently initialized and trained neural networks to quantify predictive uncertainty. Although ensembles improve prediction performance and model calibration, they induce high computational costs during training. Gal and Ghahramani (2016) propose Monte Carlo dropout to approximate predictive uncertainty with a single network trained with dropout. At inference, multiple forward passes with independently sampled dropout masks are performed to compute predictive uncertainty. Although more compute-efficient at train time, Monte Carlo dropout produces overconfident predictions compared to ensembles (Beluch et al., 2018b). More recently, Sensoy et al. (2018) proposed evidential deep learning for image classification to predict uncertainty using a single forward pass. As evidential deep learning performs on par with ensembles while drastically reducing online compute requirements, we adapt the evidential deep learning framework to our semantic segmentation task using the predictive uncertainties for label post-processing, facilitating deployment on compute-constrained robots. We use the network’s uncertainty to correct its prediction about the weeds, which is the most under-represented class and, thus, the most uncertain for the model.

Our approach combines a heuristic-based method to automatically generate partial but consistent per-pixel semantic labels. In contrast to learning-based approaches, our approach does not require human-labeled data and, at the same time, improves label consistency and, thus, the network’s prediction performance over previous heuristic-based approaches.

## 3 Materials and methods

We propose a heuristic-based approach to automatically semantically segment RGB images of agricultural fields collected using unmanned ground vehicles (UGVs) or unmanned aerial vehicles (UAVs) in three classes: soil, crop, and weed. Based on the robot’s pose, we fuse each generated semantic image label in an online-built global semantic field map. A key aspect of our approach is that we enforce spatial label consistency based on the global semantic field map. To reduce the possibility of labeling errors, we only label the detected rows as crops and the vegetation components that are far away from the rows as weeds. In this way, we trade off label quality with quantity to improve prediction performance after training our uncertainty-aware semantic segmentation network (Sensoy et al., 2018) on labels extracted from the global semantic field map. At inference time, we post-process the network’s predictions using their associated uncertainty to refine uncertain vegetation predictions.

### 3.1 Semantic field mapping

**FIGURE 2:**
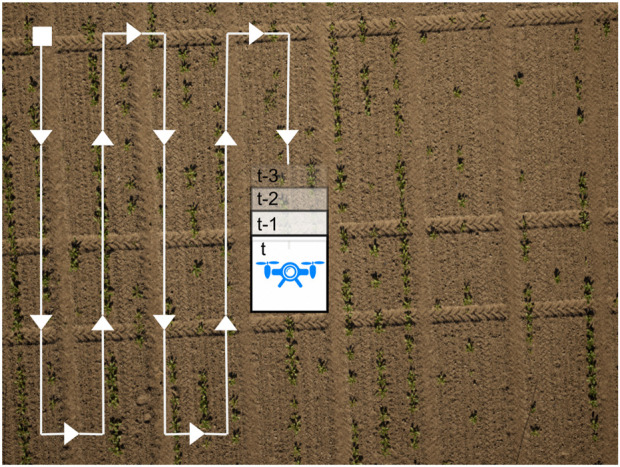
Example of a typical UAV mission. The coverage path along which we fuse semantic image labels is depicted in white, the square is the initial pose, and the arrows indicate the direction of movement. The images can overlap, but it is not required. This path maximizes the crop field coverage and is typically used in aerial data collection missions.

We perform semantic mapping to enforce spatial consistency across automatically generated semantic labels. Furthermore, the semantic map allows us to extract image-label pairs from the map with different rotations, positions, and scales. We assume that our robotic system is equipped with a downwards-facing RGB camera. At each time step t , it collects an image $I_{t}\in R^{H\times W\times 3}$ , where *H* and *W* are the height and width of the image, respectively. Let $p_{t}={\left(x_{t},y_{t},z_{t},\phi_{t}\right)}^{⊤}$ be the robot pose, where we consider the 3D position $\left(x_{t},y_{t},z_{t}\right)$ and the yaw angle $\phi_{t}\in \left(-\pi ,\pi \right]$ with respect to the origin of the mapping mission. Any path is defined by a sequence of poses that we use to fuse our predicted labels in the global semantic field map $S_{t}:G\to N^{K\times \hat{H}\times \hat{W}}$ , where G is a grid discretizing the environment into $\hat{H}\times \hat{W}$ cells with K possible semantic classes. Each image $I_{t}$ along the path is segmented by our approach based on the previous map $S_{t-1}$ and then fused into the semantic map to compute $S_{t}$ accumulating predictions. We use majority voting to assign the most likely class. In practice, we follow a common lawnmower-like coverage path to efficiently cover agricultural fields (Höffmann et al., 2023), as shown in Figure 2.

### 3.2 Automatic labeling

**FIGURE 3:**
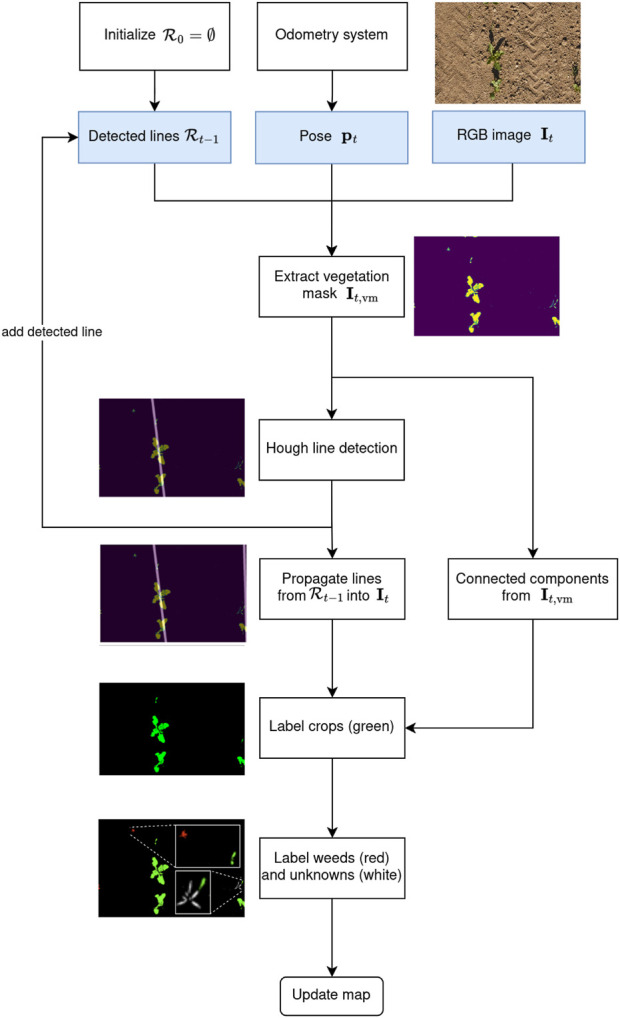
Flowchart of our automatic labeling approach with an example image. At time step t , we take as input the RGB image $I_{t}$ recorded from pose $p_{t}$ and the set of previously detected lines $R_{t-1}$ , depicted in blue boxes. First, we extract the vegetation mask $I_{t,\text{vm}}$ using a graph-segmentation approach (Felzenszwalb and Huttenlocher, 2004). Based on $I_{t,\text{vm}}$ , we compute the connected components to extract plant instances and compute the most prominent crop row via the Hough transform. We propagate the set of previously detected crop rows $R_{t}$ into the current image $I_{t}$ to track multiple crop rows. The newly detected crop row in $I_{t}$ is added to $R_{t}$ . Then, we label all connected components in $I_{t,\text{vm}}$ that intersect one of the crop rows in $R_{t}$ as crops. Furthermore, we check the distance of all other vegetation components to their closest crop row in $R_{t}$ and assign them to the weed class if their distance is above a certain threshold. Vegetation components which are too close to detected crop rows are assigned an “unknown” class that is ignored during network training to minimise labeling errors and thus maximise prediction performance.

At each time step t , our automatic labeling approach takes as input the image $I_{t}$ and the semantic field map $S_{t-1}$ to produce a semantic label for image $I_{t}$ . We use the map $S_{t-1}$ to estimate potential weeds and crops in image $I_{t}$ to enforce spatial consistency and reduce labeling errors. Our automatic labeling procedure is exemplarily visualized in Figure 3 and consists of the following steps: first, we extract the vegetation mask and apply the Hough transform to detect the main crop row in the current image $I_{t}$ . Second, we propagate all previously detected lines $R_{t-1}$ to the current pose to segment multiple crop rows. Third, we label the vegetation components with a minimal distance to all rows as weeds.

#### 3.2.1 Hough transform

**FIGURE 4:**
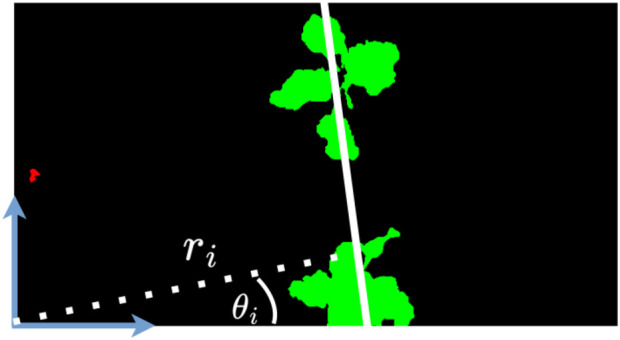
Given the vegetation mask, we visualize the line detected by the Hough transform (in white). Considering the origin as the bottom left corner, we show the parameters $r_{i}$ and $\theta_{i}$ defining the detected lines. The vegetation components intersecting the line are thus labeled as crop (green). We can see a weed (red) on the left of the image, since the vegetation component is far away from the detected line.

We compute a binary vegetation mask $I_{t,\text{vm}}\in {\left\{0,1\right\}}^{H\times W}$ using graph-based segmentation proposed by Felzenszwalb and Huttenlocher (2004), where a pixel is 1 if it contains vegetation, i.e., crop or weed, and 0 if it contains soil. We apply the Hough transform introduced by Hough (1959) to the vegetation mask $I_{t,\text{vm}}$ to detect crop rows in image $I_{t}$ . This gives us a set of supporting lines in $I_{t}$ . Each line i is parameterized by the distance $r_{t,i}$ from the image origin to the closest point on the line, and the angle $\theta_{t,i}$ between the image’s x-axis and the line connecting the origin to the closest point on the line. The origin is the lower-left pixel of $I_{t}$ . The best-fitting line is the one that maximizes the overlap with the vegetation mask $I_{t,\text{vm}}$ . In Figure 4, we show an example of a fitted crop row line (white). We discretize the Hough line radius search space using a pixel resolution of $l_{w}=5$ px to robustly fit lines in presence of noisy vegetation masks. We define the minimum number of overlapping pixels $\tau_{px}=H$ to fit the line along the whole image height. We keep only the best-fitting line of parameters $\left(r_{t},\theta_{t}\right)$ returned from the Hough Transform and add it to the set of the crop rows detected in the map $R_{t}=R_{t-1}\cup \left(r_{t},\theta_{t}\right)$ to use them in the following step. Based on the best-fitting line parameters $\left(r_{t},\theta_{t}\right)$ , we create a binary mask $I_{t,\text{line}}$ , which is 1 for all pixels on the line and 0 otherwise. We save the line mask to facilitate the computation of the following steps. The mask obtained from our example image is shown on top of the vegetation mask in Figure 3. We transform the line parameters for this time step t into the coordinate system of the mapping mission’s origin $p_{0}$ .

#### 3.2.2 Propagating predictions

We use our semantic map $S_{t-1}$ to retrieve the predicted lines $R_{t-1}$ and propagate them into our current image $I_{t}$ . This allows us to predict multiple crop rows consistent with the rows detected in previously explored areas of the crop field. At the first time step t=0 , the semantic map and $R_{0}$ are both empty, thus we skip this step. At each time step t≥1 , we compute the position of the newly acquired image in the coordinate system of the initial pose $p_{0}$ , given by the transformation matrix $T_{t}^{0}\in R^{3\times 3}$ . Then, we check which lines in $R_{t-1}$ intersect $I_{t}$ and should be propagated into its semantic prediction.

For each line i in $R_{t-1}$ , we compute the parameters $r_{t,i}$ and $\theta_{t,i}$ in the coordinate system of $p_{0}$ as.

$$r_{t,i}=\left|\left|{\left(T_{t}^{0}\right)}^{-1}\left[\begin{array}{c} r_{t-1,i}\cos\left(\theta_{t-1,i}\right)\\ r_{t-1,i}\sin\left(\theta_{t-1,i}\right)\\ 0 \end{array}\right]\right|\right|2,$$

$$\theta_{t,i}=\theta_{t-1,i}-\phi_{t},$$

Where $r_{t-1,i}\cos\left(\theta_{t-1,i}\right)$ and $r_{t-1,i}\sin\left(\theta_{t-1,i}\right)$ represent the (x,y) coordinates of the closest pixel to the origin for line i , assuming flat terrain. We include these lines in $I_{t,\text{line}}$ , i.e., we set the pixels covered by these lines to 1. To reduce the computation time, we reject lines that are too close to those already present in the mask $I_{t,\text{line}}$ . In particular, we reject line i if its distance to any other line in $R_{t}$ is smaller than $2l_{w}$ . In Figure 3, we showcase line propagation from a previous image, enabling us to detect a second crop row on the image’s right side.

As we propagate our line predictions from previously recorded images into the current image, we use an eroded version of the vegetation mask $I_{t,\text{vm}}$ to extract single vegetation components. We use a square kernel of size 3 for the erosion to remove noise from $I_{t,\text{vm}}$ and reduce the mislabeling of weeds touching the crops in the crop row. Then, all vegetation components intersecting lines in $I_{t,\text{line}}$ are assigned to the crop class, yielding a new binary mask $M_{t}\in {\left\{0,1\right\}}^{H\times W}$ where a pixel is 1 if it is labeled as crop, and 0 otherwise. We show the result in Figure 3, where soil is depicted in black and crop is depicted in green. Next, we describe which remaining vegetation components are assigned the weed class.

#### 3.2.3 Weed labeling

Naively classifying any vegetation component in $I_{t,\text{vm}}$ not yet labeled as crop in $M_{t}$ usually results in poor weed label quality. Although these remaining vegetation components might be crop, the row detection could have failed because of low sensor resolution, wrong odometry or pose information, or bad lighting conditions (Lottes et al., 2016), such that these crop instances are not included in $M_{t}$ . To avoid labeling these potential crops as weeds, we do not label the vegetation components, which are likely to introduce labeling errors and ignore them during network training. To this end, we compute the distance from each of the N crop pixels of $M_{t}$ with value 1 to their respective closest line as follows

$$d\left(x,y\right)={argmin}_{\left(r_{t,i},\theta_{t,i}\right)\in R_{t}}\;|x\cos\left(\theta_{t,i}\right)+y\sin\left(\theta_{t,i}\right)-r_{t,i}|.$$

We aim to estimate crop sizes along the detected rows using these distances d(x,y) . Hence, we use an indicator function 1(x,y) that returns 1 if the pixel (x,y) is 1 in $M_{t}$ and zero otherwise to extract the mean $\mu_{d}=\frac{\sum_{\left(x,y\right)}1\left(x,y\right)\;d\left(x,y\right)}{N}$ and standard deviation $\sigma_{d}=\sqrt{\frac{\sum_{\left(x,y\right)}1\left(x,y\right)\;{\left(d\left(x,y\right)-\mu_{d}\right)}^{2}}{N}}$ . We define the minimum distance $d_{\text{min}}$ required for any unlabeled vegetation instance to be labeled as a weed as

$$d_{\text{min}}=\mu_{d}+\delta \;\sigma_{d},$$

where δ=3 in our setting, such that only vegetation instances with a large distance from all rows are considered weeds. All vegetation components that were not labeled as crops and whose distance to the lines is smaller than $d_{\text{min}}$ are left unlabeled. Note that large values of δ reduce the number of components labeled as weeds, while small values of δ are prone to weed labeling errors. The key idea behind this step is that $\mu_{d}$ and $\sigma_{d}$ represent the area around the detected crop row where we assume there may be other crops that were not touched by the line and that we leave unlabeled. Outside of this area, we are fairly confident that the vegetation component is a weed as it is far from the detected crop row with plants of estimated size $\mu_{d}$ . The resulting label for the example image is shown in Figure 3, where components close to the crop row on the right are not labeled while the component on the upper-left corner is labeled as a weed.

### 3.3 Learning with uncertainty

Once we finish our mapping mission as described in Section 3.2, we can extract any number of image-label pairs with any size, rotation, and aspect ratio. We use the extracted labels to train a semantic segmentation network. We follow the evidential deep learning framework by Sensoy et al. (2018) to predict semantic segmentation and the network’s prediction uncertainty at the same time. Estimating the prediction uncertainty allows us to account for the “unknown” class by refining the network’s semantic predictions in a post-processing step described in Section 3.4.

The key idea behind evidential deep learning is to predict a Dirichlet distribution over all possible class probabilities instead of a single point estimate as in deterministic deep neural networks. In this way, the evidential network minimizes the prediction error while maximizing the prediction uncertainty for ambiguous image parts. We use evidential deep learning instead of Bayesian deep learning approaches (Gal and Ghahramani, 2016; Beluch et al., 2018a) as it is empirically shown to produce similarly or better-calibrated prediction uncertainties (Sensoy et al., 2018) while being computationally more efficient during training than ensemble methods and during inference than Monte Carlo dropout.

We train the network to minimize the Bayes risk cross-entropy for a pixel (x,y) of image I ,

$$L_{CE,\left(x,y\right)}=\overset{K-1}{\underset{k=1}{\sum }}y_{\left(x,y\right),k}\left(\psi \left(Q_{\left(x,y\right)}\right)-\psi \left(\alpha_{\left(x,y\right),k}\right)\right),$$

where ψ is the digamma function, $y_{\left(x,y\right),k}=1$ if the pixel (x,y) of I belongs to ground truth class k , $Q_{\left(x,y\right)}=\sum_{k=1}^{K}\alpha_{\left(x,y\right),k}$ , and $\alpha_{\left(x,y\right),k}$ is the evidence predicted by the network in support of class k . We do not compute this loss for the pixels assigned to the “unknown” class, so we sum only over the remaining K−1 classes, i.e., soil, crop, and weed. We additionally minimize the Kullback-Leibler (KL) divergence between the uniform $D\left(1_{K-1}\right)$ and predicted Dirichlet distribution $D\left({\tilde{\alpha }}_{\left(x,y\right)}\right)$ for all non-ground-truth classes (Sensoy et al., 2018),

$$L_{\left(x,y\right)}=L_{CE,\left(x,y\right)}+\lambda_{\text{epoch}}KL\left[D\left({\tilde{\alpha }}_{\left(x,y\right)}\right)\|D\left(1_{K-1}\right)\right],$$ (1)

$${\tilde{\alpha }}_{\left(x,y\right),k}=y_{\left(x,y\right),k}+\left(1-y_{\left(x,y\right),k}\right)\alpha_{\left(x,y\right),k},$$

for all K−1 classes, and $\lambda_{\text{epoch}}=\min\left(1.0,\frac{\text{epoch}}{T}\right)$ with epoch being the current training epoch and T the maximum annealing epoch. We minimize the overall training loss

$$L=\frac{1}{HW}\overset{H}{\underset{x=1}{\sum }}\overset{W}{\underset{y=1}{\sum }}L_{\left(x,y\right)},$$

which is the average over all image pixels, iterating over all training images. At inference time, the network predicts the semantic class and an uncertainty for each pixel, that we use for our label refinement.

### 3.4 Uncertainty-based label refinement

We use the network’s predicted Dirichlet distribution $D\left(\alpha_{\left(x,y\right)}\right)$ over all K−1 classes to quantify the prediction uncertainty for post-processing and refining the predicted semantic labels. The network’s prediction uncertainty (Sensoy et al., 2018) for a pixel (x,y) of image I is given by

$$u_{t,\left(x,y\right)}=\frac{K-1}{\sum_{k=1}^{K-1}\alpha_{\left(x,y\right),k}},$$

where K−1 is the number of classes without the “unknown” class. In our crop-weed segmentation case, the most under-represented class is weed. Thus, the network will be more uncertain about areas representing weeds than the other classes. We define an adaptive threshold to select the most uncertain pixels (x,y) in any image I as

$$\tau =\frac{\max\left(u_{\left(x,y\right)}\right)-\min\left(u_{\left(x,y\right)}\right)}{2}+\min\left(u_{\left(x,y\right)}\right).$$

We compute a binary mask $U_{t}\in {\left\{0,1\right\}}^{H\times W}$ where a pixel (x,y) is 1, if $u_{\left(x,y\right)}>\tau$ , and 0 otherwise. We compute the connected components of our semantic prediction, aiming to use the ratio between the size of the object and its number of uncertain pixels to refine the component’s label. Most of the vegetation components have high uncertainty at their instance boundaries. Instead, we are interested in those components for which also large amounts of interior pixels are uncertain. We iterate over all c∈{1,…,C} crop components in our network’s prediction and compute for each one a binary mask $C_{c}\in {\left\{0,1\right\}}^{H\times W}$ , which is 1 for all pixels belonging to the component. We also compute their bounding box $b_{c}=\left(b_{c}^{x},b_{c}^{y},b_{c}^{\text{height}},b_{c}^{\text{width}}\right)$ , where $b_{c}^{x}$ and $b_{c}^{y}$ are the coordinates of the upper left corner of the bounding box, while $b_{c}^{\text{height}}$ and $b_{c}^{\text{width}}$ are the height and width of the bounding box. We define an adaptive threshold

$$\tau_{c}=\frac{1}{4}\min\left(\frac{b_{c}^{\text{width}}}{b_{c}^{\text{height}}},\frac{b_{c}^{\text{height}}}{b_{c}^{\text{width}}}\right).$$

This threshold helps us avoid detecting as weeds a lot of small spikes of uncertainty that could arise because of shadows, reflections, or insects. In this way, we only act upon vegetation components where there is a large uncertain area. If the network is uncertain about the prediction of crop component c , it holds that

$$\frac{\sum_{\left(x,y\right)}U_{\left(x,y\right)}C_{c,\left(x,y\right)}}{b_{c}^{\text{width}}\;b_{c}^{\text{height}}}>\tau_{c}.$$ (2)

**FIGURE 5:**
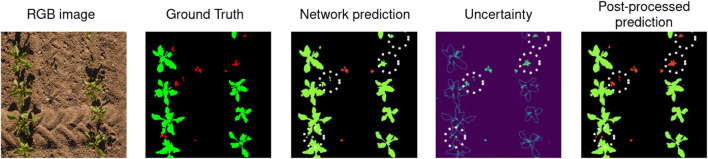
For the RGB input image on the left, we show the semantic ground truth labels, where crops are represented in green, weeds in red, and the soil in black. Then, we show our network’s prediction and we highlights some mistakes using white dotted circles, where weeds are mislabeled as crops. The fourth image shows our network’s uncertainty. As expected, the network is mostly uncertain about the boundaries of the plants and about the weeds, we see that even the weeds labeled as crops in our prediction have high uncertainty. The last image shows our post-processed prediction, after we label as weeds the highly uncertain vegetation components. We can see that this corrects many of the network’s errors.

If crop component c fullfills Equation 2, we assign the component’s uncertain pixels (x,y) with $U_{\left(x,y\right)}=1$ to the weed class. We do not re-assign the whole vegetation component as a weed because our network does not provide instances. Hence, there may be components that contain both weeds and crops. These components likely have higher uncertainty since they are labeled as “unknown” and thus being ignored during training. We show in Figure 5 the result of our post-processing operation for an example image, highlighting the correspondence between the network’s wrong predictions, the estimated uncertainty and the post-processed semantic prediction.

## 4 Results

The main focus of this work is an automatic labeling pipeline for semantic soil-weed-crop segmentation of RGB images. The results of our experiments support our key claims: our approach (i) generates more accurate semantic labels than previous unsupervised label generation approaches on multiple datasets; (ii) we outperform previous unsupervised semantic segmentation approaches by combing our spatially consistent generated labels and uncertainty-aware semantic neural networks; and (iii) we improve the performance of fully supervised models on previously unseen crops, growth stages, and soil conditions after fine-tuning the network using our automatically generated labels.

### 4.1 Experimental setup

#### 4.1.1 Datasets

We use four datasets, three of which are publicly available: PhenoBench (Weyler et al., 2024), as well as the Carrots and Onions from Lincoln University (Bosilj et al., 2020), and a Sugar Beets dataset introduced by Weyler et al. (2022b). The Carrots dataset was recorded in Lincolnshire, United Kingdom, in June. The field is under substantial weed pressure and contains weeds with a similar appearance to the crop. Furthermore, several regions of vegetation contain crops and weeds in close proximity. The Onions dataset was also recorded in Lincolnshire, United Kingdom, but in April. The weed pressure is lower compared to the Carrots dataset. The PhenoBench dataset was recorded in Meckenheim, Germany, on different dates between May and June to capture different growth stages. The field contains two varieties of sugar beets and six different weed varieties. The weed pressure varies as the dataset contains images from fields that were fully, partially, or not treated at all with herbicides. The Sugar Beets dataset was also recorded in Meckenheim, Germany, over five different weekly sessions. The field is arranged with small spacing between plants and shows high weed pressure, inducing challenging conditions. We refer to Table 1 for information about the camera, image resolution, and ground sampling distance of the datasets.

**TABLE 1:** Details for the datasets used in the paper: name, reference paper, camera sensor, image resolution and GSD.

Dataset	Reference	Camera	Image resolution [px]	GSD $\left[\frac{\text{mm}}{\text{px}}\right]$
PhenoBench	Weyler et al. (2024)	PhaseOne iXM-100 with a 80 mm RSM prime lens on a gimbal (UAV)	11664×8750	1
Carrots	Bosilj et al. (2020)	Teledyne DALSA Genie Nano deployed on a manually pulled cart (UGV)	2428×1985	0.4
Onions	Bosilj et al. (2020)	Teledyne DALSA Genie Nano deployed on a manually pulled cart (UGV)	2149×1986	0.4
Sugar Beets	Weyler et al. (2022b)	PhaseOne iXM-100 (UAV)	4320×4100	1.5

#### 4.1.2 Training details and hyperparameters

We use ERFNet (Romera et al., 2018) as our network trained using the Adam (Kingma and Ba, 2015) optimizer, a learning rate of 0.01, and a batch size of 32. We set T=25 in Equation 1 to linearly increase $\lambda_{\text{epoch}}$ over the first 25 epochs. We report all the hyperparameters of our method with their values in Table 2. To evaluate the quality of the labels, we generate labels for the validation sets of PhenoBench and Sugar Beets, as well as for the whole Carrots and Onions dataset. Second, we automatically generate labels for the images in their training sets to train our network and evaluate the results on the manually annotated validation sets. We do not split Carrots and Onions to train on them since they consist of only 20 images each. Thus, we do not use them for model training. Instead, we evaluate our label generation and the generalization capabilities of fine-tuned models on these datasets.

**TABLE 2:** List of the hyperparameters of our method, where they are used, and their chosen values.

Hyperparameter	Method	Value
Minimum number of pixels for detection $\left(\tau_{px}\right)$	Hough line detection	H (i.e., image height)
Width of the line to fit $\left(l_{w}\right)$	Hough line detection	5 px
Confidence interval for crop rows (δ)	Weed labeling	3
Maximum number of annealing epochs (T)	Evidential deep learning	25

#### 4.1.3 Metrics

We use the intersection over union (IoU) (Everingham et al., 2010) as a metric for all of our experiments. For the automatic labeling pipeline, we also report the boundary IoU (Cheng et al., 2021) to have a better understanding of the approaches’ limitations. The reported mean IoU (mIoU) values are the macro-averages over all classes.

#### 4.1.4 Baselines

We use three baselines: two are general-purpose unsupervised semantic segmentation networks not specifically developed for the agricultural domain, while one is an automatic labeling method specifically developed for the agricultural domain. The first baseline is STEGO by Hamilton et al. (2022), which provides an official implementation for the evaluation alongside their models. We use the model trained on MS COCO (Lin et al., 2014) with the vision transformer architecture (Dosovitskiy et al., 2021). STEGO predicts different per-pixel features and then clusters them using self and cross attention mechanisms (Vaswani et al., 2017). Our second baseline is U2Seg by Niu et al. (2024), which builds on top of STEGO and uses instance information to overcome some of the limitations of the previous work; they also open-source their code and provide their models. U2Seg proposes a universal segmentation, coupling instances and semantic classes at training time, to predict clusters at inference time for which they recover class and instance labels. We use the model trained on Imagenet (Deng et al., 2009) and MS COCO with 800 clusters. Lottes et al. (2016) propose a domain-specific method for generating per-pixel crop and weed labels. They use a vegetation mask to detect the main crop row and then label all other vegetation components as weeds. We use their official implementation, removing the NIR image channels. We evaluate their automatically generated labels (base) and the performance of ERFNet trained on their labels (learned). We train the same network with the same training hyperparameters on their and our generated label to ensure a fair comparison. We report the results of ERFNet trained on the manually annotated training set of PhenoBench and evaluated on the validation set as an upper performance bound.

### 4.2 Automatic labeling

In the first experiment, we show that our automatic labeling pipeline generates more accurate semantic soil-weed-crop labels than other methods on multiple datasets. We compare against two general-purpose unsupervised semantic segmentation networks and the domain-specific approach by Lottes et al. (2016).

We show the results on all four datasets in Table 3. The general-purpose approaches perform worse than the domain-specific methods across all datasets, except for U2Seg on the Onions dataset. As Onions have thin leaves, they are hard to detect with common color histogram thresholding methods, such as the one by Lottes et al. (2016). Furthermore, the weeds in this dataset are the same size as the crops, leading to bias in crop row detection and introducing a higher risk of confusing weeds and crops. Our approach for label generation, referred to as Ours (base), shows higher crop label quality than Lottes (base) while performing on par or better in terms of weed label quality. Particularly, Lottes (base) confuses substantially more weeds with crops, while our approach, by design, does not assign labels to hard-to-label vegetation components, as described in Section 3.2. The Carrots dataset is the only one where U2Seg outperforms the domain-specific approaches, which suffer from the weed pressure when estimating the crop rows. Our method consistently outperforms all other baselines across all datasets with different crop species, weed pressure, growth stages and lighting conditions. Most approaches fail on the Onions dataset due to brighter illumination and thin crops. In contrast, our approach improves by approx. 9% mIoU over the second-best baseline, U2Seg, importantly showing highest improvements in both vegetation classes.

**TABLE 3:** Performance of all the baselines on the PhenoBench dataset, Carrots dataset, Onion dataset, and Sugar Beets dataset.

Dataset	Approach	IoU [%]			mIoU	Boundary IoU [%]		
		Soil	Crop	Weed		Soil	Crop	Weed
PhenoBench	STEGO	21.4	11.9	0.4	11.2	0.0	1.5	0.0
	U2Seg	84.6	40.0	2.4	42.3	45.8	11.7	3.4
	Lottes (base)	**99.6**	44.1	**7.6**	50.5	0.0	0.0	0.9
	Ours (base)	98.8	**80.7**	7.2	**62.2**	**86.3**	**79.1**	**13.2**
Carrots	STEGO	28.4	5.1	15.8	16.4	0.0	0.9	0.0
	U2Seg	80.1	**20.4**	2.3	34.3	36.2	0.0	**19.3**
	Lottes (base)	89.1	15.9	34.0	46.3	0.0	0.0	6.8
	Ours (base)	**90.4**	12.6	**42.7**	**48.6**	**84.4**	**23.6**	9.4
Onion	STEGO	26.5	5.1	3.0	11.5	0.0	2.4	0.0
	U2Seg	92.8	0.0	4.3	32.4	24.2	0.0	8.2
	Lottes (base)	89.7	1.4	1.1	30.7	0.0	0.0	1.6
	Ours (base)	**95.4**	**10.7**	**16.6**	**40.9**	**74.2**	**10.7**	**16.7**
Sugar Beets	STEGO	24.9	4.7	1.3	10.3	0.0	1.9	0.0
	U2Seg	77.9	9.9	6.7	31.5	1.8	**2.8**	0.0
	Lottes (base)	**98.0**	23.6	18.8	46.8	0.0	0.0	1.5
	Ours (base)	97.7	**50.6**	**24.7**	**57.7**	**88.7**	0.0	**1.8**

The top rows are the general purpose approaches, while the bottom rows are the domain-specific ones. We report the mean IoU, plus the IoU and boundary IoU per class. In bold the best results per column.

**FIGURE 6:**
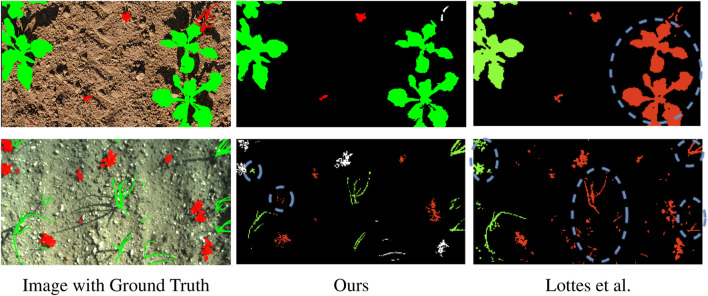
Qualitative results of our and Lottes et al. methods on PhenoBench (top row) and Onions (bottom row). Soil is black, crops are green, weeds are red, vegetation that we leave unlabeled is white. In the dashed blue circles, we highlight segmentation errors.

The boundary IoU confirms the result of the standard IoU metric. As shown in Table 3, the approach by Lottes et al. (2016) poorly segments boundaries on most of the datasets. This might be due to wrongly segmented vegetation masks. Aiming to include the boundary of weeds more accurately may worsen the overall performance since soil could be wrongly considered as vegetation. We hypothesise that our approach might suffer from the same problem on the Carrots dataset. The difference between IoU and boundary IoU per class suggests that we underestimate the size of weeds, i.e., high IoU but low boundary IoU for weeds, and overestimate crop size, i.e., low IoU but high boundary IoU for crops. On the Carrots dataset U2Seg outperforms the other methods on the weeds boundary IoU. The weed IoU suggests that U2Seg overestimates weeds, thus obtaining a boost as the total number of pixels in the IoU computation is low. On the Onions dataset, our method’s IoU and boundary IoU are almost the same irrespective of the semantic class since the crops and weeds are thin. Thus, the boundary area covers the whole vegetation instance. The other approaches fail to correctly assign weed and crop boundaries on the Onion dataset, which follows from the low weed and crop IoU. On the Sugar Beets dataset, all approaches fail to predict boundaries, most likely due to unusually high weed pressure. Our method accurately segments soil boundaries, suggesting that it at least successfully differentiates between soil and vegetation. Overall, the results suggest that most approaches underestimate the size of vegetation, both crops and weeds. Instead, our automatic labeling method shows the strongest boundary segmentation performance across all methods and classes on most datasets, often by a large margin compared to the second-best method. This further verifies our claim that our automatic labeling pipeline generates more accurate semantic soil-weed-crop labels than previous methods. We show qualitative results of Lottes et al. (2016) and our approach in Figure 6.

### 4.3 Unsupervised semantic segmentation

The second experiment evaluates the performance of our automatic label generation combined with network training and uncertainty post-processing on the PhenoBench dataset. We show that training the evidential ERFNet using our automatically generated labels outperforms other unsupervised semantic segmentation models. The general-purpose learning-based approaches have not been fine-tuned on human-labeled field images to ensure a fair comparison. Our approach and Lottes et al. (2016) generate labels on the PhenoBench training set. We use the public training set of images to have a fair comparison with the fully suprvised ERFNet model, trained on the manual labels. Trained models are evaluated on the official PhenoBench validation set.

Table 4 summarizes the results. We use (learned) to refer to the results obtained by ERFNet after being trained on the labels generated by the approach, and we use (+uncertainty) to refer to the previous results once we post-process them using the uncertainty estimated by the model. The approach by Lottes et al. (2016) confuses more crops with weeds since it naively assigns all vegetation components that are not on the main crop row to the weed class. Hence, Lottes et al. (2016) introduce inconsistent labels in the model’s training data. Thus, training the ERFNet on Lottes’ labels does not yield uncertainty estimations that are useful for improving the predictions during post-processing. Additionally, this leads to smaller performance improvements after training on their labels than after training on our labels. Using our generated labels to train the ERFNet substantially improves the weed and crop predictions over directly using our generated labels. We further improve mIoU and weed predictions by exploiting the estimated uncertainties in Ours (uncertainty) for post-processing. Most importantly, Ours (uncertainty) noticeably closes the performance gap between fully supervised and state-of-the-art unsupervised approaches. However, the ERFNet trained on human-labeled training images still predicts weeds more accurately. As the fully supervised model predicts more weeds, it also confuses weeds with crops more often. Hence, our approach performs better on both the crop and soil classes. This experiment confirms that our method’s conservative approach to labeling, ignoring vegetation components likely to introduce labeling errors combined with evidential deep learning, is a viable solution to largely reduce the need for manually annotated images.

**TABLE 4:** Performance of ERFNet trained on the labels generated by ours and the approach by Lottes et al.

Approach	IoU [%]			mIoU
	Soil	Crop	Weed	
Lottes et al. (learned)	**99.1**	54.6	11.2	55.0
Lottes et al. (+uncertainty)	**99.1**	27.2	8.1	44.8
Ours (learned)	**99.1**	**88.8**	21.0	69.6
Ours (+uncertainty)	**99.1**	88.6	**22.7**	**70.1**
Ours (PhenoBench test)	99.5	87.9	24.6	70.7
ERFNet (fully supervised)	98.0	83.4	33.5	71.6

We also report the results when we use the uncertainty to post process the semantic predictions. The bottom line shows a fully supervised approach trained on manual labels as upper bound of the performance. Best results per column in bold.

### 4.4 Generalization capability

In the third experiment, we show that our approach enhances the performance of networks trained in a fully supervised fashion by fine-tuning on unseen fields using our automatically generated labels. We do not use our evidential network but train an ERFNet using the standard cross-entropy loss to seamlessly fine-tune existing networks pre-trained in a fully supervised fashion. We train two ERFNets, one on each of the human-labeled training sets of PhenoBench and Sugar Beets. We deploy the two models on all four datasets without fine-tuning. Then, we fine-tune the two models leveraging our automatically generated labels for the Sugar Beets and PhenoBench datasets. Each model is fine-tuned on the dataset that it was not trained on.

In Table 5, we show the performance of the two models. In brackets, we provide the performance difference after fine-tuning, where blue and red indicate performance improvements or degradations, respectively. The gray rows show the models’ performances on the dataset they were trained on. Due to the domain gap between datasets, the models that were not fine-tuned have a lower performance when evaluated on unseen data. Fine-tuning the models makes the performance over the original training data worse as they aim to learn features that are common to both datasets. Our results suggest that using our automatically generated labels helps to close the performance gap on previously unseen datasets with different crops, soil types, lighting conditions, and sensor setups. Generally, our fine-tuned models perform better on all classes and datasets, even on the Onions and Carrots datasets, the model was not pre-trained nor fine-tuned on. Only the model that is fine-tuned on the Sugar Beets dataset does not improve performance on the Carrots dataset. We hypothesize this is because the PhenoBench dataset is approx. 10× larger than Sugar Beets introducing data imbalance while automatically generated Sugar Beets labels are of lower quality than labels generated on PhenoBench. In sum, using our automatically generated labels helps to fine-tune fully supervised models, enabling better adaptation to unseen field conditions without any additional human labeling costs.

**TABLE 5:** Performance of fully supervised models trained on manually annotated data, and in brackets the difference with respect to the model after fine-tuning.

Train		Test	IoU [%]						mIoU	
			Soil		Crop		Weed			
PhenoBench	(+ Sugar Beets)	PhenoBench	98.0	(−0.4)	83.4	(−11.0)	33.5	(−11.7)	71.6	(−7.7)
		Sugar Beets	93.5	(+0.2)	7.3	(+44.4)	16.8	(+8.2)	39.2	(+17.6)
		Carrots	89.0	(−2.5)	11.1	(+14.9)	47.1	(−11.7)	49.1	(+0.2)
		Onions	82.4	(+5.3)	0.5	(+5.0)	11.3	(−4.4)	31.4	(+2.0)
Sugar Beets	(+PhenoBench)	PhenoBench	97.6	(−0.1)	67.0	(+9.8)	11.7	(+4.7)	60.2	(+3.4)
		Sugar Beets	98.3	(−4.2)	72.4	(−10.9)	59.2	(−20.5)	76.6	(−11.8)
		Carrots	87.6	(+1.0)	36.1	(+2.1)	24.3	(+10.0)	49.0	(+4.7)
		Onions	86.3	(+1.0)	0.2	(+12.1)	13.2	(+0.7)	33.2	(+4.6)

In red if the fine-tuned model performs worse, in blue if it performs better. The gray cells show the performance on the same dataset.

## 5 Discussion

A robust perception system is crucial for the successful deployment of robotic platforms in arable fields. Most perception systems rely on data-driven machine learning approaches to train vision models that automatically interpret the data collected with onboard sensors, such as RGB cameras. Thus, reliable and accurate learning-based perception systems are crucial to providing valuable information to farmers or autonomous robots. Most learning-based semantic segmentation approaches assume access to large amounts of human-labeled data required to train the vision model. However, their performance rapidly decreases in field conditions they were not trained on, i.e., different crop species, growth stages, weed pressure, and lighting conditions.

To address this issue, we proposed an automatic labeling approach to obtain semantic information from RGB images of agricultural fields. Our method shows semantic segmentation performance close to the performance of a model trained on large amounts of human-labeled data in a fully supervised fashion. This significantly reduces the need for manually annotated data, reducing costs and relaxing the need for domain experts. The arable field dataset works considered in our experimental evaluation report an average of 2 h *per image* for labeling the Onions dataset, 3–4 h *per image* for the Carrots dataset, and 1–3.5 h *per image* for the PhenoBench dataset. All of the datasets went through at least two labeling rounds, doubling the costs. This highlights the need for new labeling paradigms beyond fully supervised model training while maintaining strong prediction performance. Our method is a crucial step towards closing the performance gap between models trained in an unsupervised fashion and fully supervised models without adding additional labeling costs.

In our experiments, we show that the fully supervised approach has a lower performance in segmenting crops compared to our unsupervised method, as it is trained on more weed instances. Nevertheless, the fully supervised method still shows the highest mIoU. The unsupervised methods are not exposed to enough weed labels, making them assign the crop class more often. Since the number of crop pixels is generally higher, these errors have a smaller impact on the crop than on the weed segmentation. We also investigate how to use our automatic labeling in combination with supervised methods to improve the overall performance in challenging scenarios, i.e., in unseen fields with new crop species and different weed pressure. Fine-tuning comes at the cost of performing worse on the pre-training dataset, as shown in Table 5. The degradation largely depends on the size and similarity of the pre-training and automatically labeled dataset used for fine-tuning. Future work could investigate continuous learning methods to train on the newly automatically labeled images without catastrophically forgetting what has already been learned.

The need for posed images can be a limitation of our method as it cannot be applied to a dataset of unposed images. However, most of the agricultural datasets are recorded using aerial or ground vehicles that, by default, provide spatial information while recording images in the field, often using GNSS systems such as GPS. Furthermore, we assume deployment in a managed agricultural field. If this assumption does not hold and the weeds are larger than the crops, our crop row detection fails and leads to degraded results. Our results, as well as those by Lottes et al. (2016), show that we could make use of a better vegetation mask to improve unsupervised methods. One possible solution would be to use NIR images, which are less dependent on the lighting conditions compared to RGB images. NIR images are already commonly used for crop segmentation in agriculture (Sahin et al., 2023; Colorado et al., 2020). Moreover, our approach leverages uncertainty estimates to post-process semantic predictions. Current state-of-the-art methods are known to produce partially miscalibrated uncertainty estimates (Beluch et al., 2018a). Thus, our post-processing could benefit from improvements in uncertainty-aware deep learning. Finally, we plan to deploy our approach on a real robot to perform field trials.

## 6 Conclusion

In this paper, we presented a novel approach to automatically generate semantic soil-crop-weed labels of images from agricultural fields. We evaluated our approach on four datasets recorded with different robotic platforms and in various fields. Our approach outperforms previous domain-agnostic and domain-specific unsupervised labeling approaches. Furthermore, we showed that our generated labels allow fine-tuning networks trained in a fully supervised fashion on one dataset to other agricultural fields, e.g., different species, growth stages, and field conditions. In this way, our approach increases the semantic segmentation generalization capabilities of existing networks for soil-weed-crop segmentation without additional human labeling effort.

## Data Availability

The original contributions presented in the study are included in the article/supplementary material, further inquiries can be directed to the corresponding author.
